# Cytomorphological Effects of Lightweight and Heavyweight Polypropylene Mesh on the Ilioinguinal Nerve: An Experimental Study

**DOI:** 10.7759/cureus.37038

**Published:** 2023-04-02

**Authors:** Betül Keskinkılıç Yağız, Ebru Esen, Cihangir Akyol, İlknur Kepenekçi Bayram, Oya Evirgen, Can Ateş, Ercüment Kuterdem

**Affiliations:** 1 General Surgery, Samsun Gazi State Hospital, Samsun, TUR; 2 Surgical Oncology, Ministry of Health University Gulhane Educational and Research Hospital, Ankara, TUR; 3 Department of General Surgery, Ankara University School of Medicine, Ankara, TUR; 4 Department of Histology and Embryology, Ankara University School of Medicine, Ankara, TUR; 5 Department of Biostatistics, Aksaray University School of Medicine, Aksaray, TUR

**Keywords:** postoperative pain, ilioinguinal nerve, lightweight mesh, heavyweight mesh, inguinal hernia repair

## Abstract

Objective

This study aimed to investigate the cytomorphological effects of heavyweight and lightweight mesh on the ilioinguinal nerve in an experimental animal model.

Methods

Sixteen New Zealand male rabbits were included in the study. The left inguinal regions of the first six animals were assigned as controls and the right inguinal regions were assigned as the sham group. The left inguinal regions of the remaining 10 animals were assigned as the lightweight mesh group and the right inguinal regions were assigned as the heavyweight mesh group. No intervention was performed in the control group. In the sham group, only ilioinguinal nerve exploration was performed. In mesh groups, ilioinguinal nerve exploration was performed and the mesh was implanted on the ilioinguinal nerve. After three months, ilioinguinal nerve specimens were excised from both sides for cytomorphological examination.

Results

Myelin sheath thickening, separation of the myelin layers, and myelin vacuolization were more pronounced in the heavyweight mesh group compared to the lightweight mesh group. The G-ratio was moderately increased in the heavyweight mesh group when compared to other groups. The ratio of fibers with ≤4 µm diameter was higher in the lightweight mesh group compared to other groups, and the ratio of fibers with ≥9 µm diameter was higher in the heavyweight mesh group than in the other groups (p<0.05).

Conclusion

Both of the meshes induce cytomorphological alterations on the adjacent nerve tissues caused by foreign body reaction and compression. Ilioinguinal nerve degeneration was more pronounced in the heavyweight mesh than in the lightweight mesh. Histological alterations on the ilioinguinal nerves caused by different meshes may be related to chronic pain after hernia surgery. We believe our study will serve as a guide for future studies on the topic.

## Introduction

Hernia repair is one of the most commonly performed surgical procedures in general surgery practice and is associated with substantial health costs. Inguinal hernias account for 75% of all hernia cases [1]. It has been estimated that the lifetime risk of developing an inguinal hernia is 27% in males and 3% in females, and more than 20 million hernia repair procedures are performed worldwide annually. Groin hernia may present with complications that may lead to mortality and morbidity such as incarceration and strangulation. It has been shown that overall and major morbidity and mortality rates in patients who underwent emergency surgery for incarcerated inguinal hernia were 41.5%, 9.6%, and 3.4%, respectively [2].

Conventional techniques used in inguinal hernia repair have been replaced with surgical procedures involving laparoscopic surgery and different meshes [3]. Complications such as hematoma, surgical site infection, urinary retention, recurrence, and chronic pain have been reported during short or long-term follow-ups after inguinal hernia repair [4,5]. Despite the widespread use of mesh grafts and associated successful outcomes, efforts to design and develop new meshes that are more comfortable and more compatible with the organism are ongoing. Besides, properties of meshes including pore size, geometry, active surface area, affinity to water, elasticity, polymer type, and biocompatibility have also received significant attention [6].

Heavyweight meshes have thick polymer fibers, small pores, high tensile strength, and large surface area and they provide maximum scar tissue and optimal mechanical stability. On the other hand, lightweight meshes have thin polymer fibers, large pores, and small surface area and they allow for high elastic repairs that are more compatible with the tissue in the long term by leading to lower incidences of scar tissue [7]. It remains unclear whether the prosthetic meshes have an unfavorable (structural or functional) impact on the neurovascular structures in the inguinal canal, the ductus deferens, and the adjacent vascular and neural structures due to direct mesh contact or fibrosis developed around the mesh.

The rate of postoperative chronic pain following inguinal hernia surgery, which significantly impairs patients’ daily activities and quality of life, has been reported to be between 15 and 53% [8]. The association of postoperative chronic pain with surgical techniques and materials used is still under debate. The risk factors proposed to be related to chronic pain include the experience of the surgeon, surgery for recurring hernia, nerve injury, mesh implantation, type of surgery, psychosocial factors, presence of preoperative pain, patients’ age, early-onset pain after surgery, and ischemia [8,9]. A recent study found a lower incidence of chronic inguinal pain after laparoscopic repair than after open hernia repair [10]. Although laparoscopic repair is related to a lower incidence of chronic pain, open repair remains the common technique of choice. A recent study reported that open repair was performed in 28% of patients who underwent inguinal hernia repair in a one-year period [11].

Little is known about the underlying pathophysiological mechanisms and neurophysiological changes related to chronic pain encountered after hernia repair. The relationship between postoperative chronic pain and sensory impairment supports the hypothesis that the pain is neuropathic [12]. Postoperative chronic pain may occur due to injury to one or more nerves (ilioinguinal, iliohypogastric, and genitofemoral nerves) in the inguinal region. This injury may result from tissue damage during incision or from dissection and fixation procedures during mesh implantation. Moreover, postoperative inflammation or mesh reaction may also contribute to this injury [12]. These nerves need to be protected during surgery to prevent postoperative pain and sensory loss. This study aimed to investigate the cytomorphological effects of heavyweight and lightweight meshes on the ilioinguinal nerve during open hernia repair.

## Materials and methods

Subjects

Sixteen male New Zealand rabbits with ages ranging from six months to one year and weighing 2.5-3 kg were included in the study. Before enrollment, the animals were monitored for three weeks to provide a quarantine period and for getting adapted to their new environment. The animals were kept in cages on a 12-hour light/dark cycle at an environmental temperature of 22 ºC and were exposed to a standard feeding procedure with tap water during the experimental period. Subjects were randomly divided into two groups initially. The left inguinal regions of the first six animals were assigned as controls and the right inguinal regions were assigned as the sham group. The left inguinal regions of the remaining 10 animals were assigned as the lightweight mesh group and the right inguinal regions were assigned as the heavyweight mesh group. This study, which was conducted in the Animal Laboratory of Ankara University Medical Faculty, was approved by the Experimental Animals Ethics Committee of Ankara University (61-312; 14 April 2010). Surgical procedures were performed in accordance with the European Convention for the Protection of Vertebrate Animals Used for Experimental and other Scientific Purposes [13].

Surgical procedure

**Figure 1:**
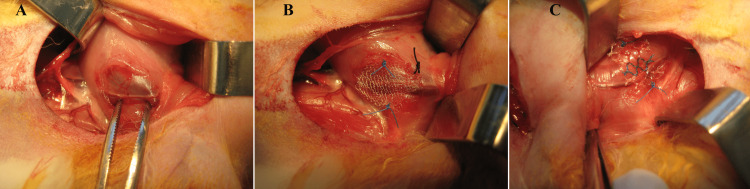
Exposure of the ilioinguinal nerve and mesh placement (A) ilioinguinal nerve; (B) heavyweight mesh placement; (C) lightweight mesh placement

All surgical procedures were performed by two senior general surgery residents (first and second coauthors of the manuscript) at the same time of the day. After a 12-hour fasting period, 50 mg/kg cefazolin sodium was administered intramuscularly (Cefamezin®, Eczacibasi Pharmaceuticals, Istanbul, Turkey). For the induction of anesthesia, a mixture of 35 mg/kg ketamine hydrochloride (Ketalar®, Pfizer, Istanbul, Turkey) and 5 mg/kg xylazine hydrochloride (Rompun® 2%, Bayer, Istanbul, Turkey) was administered intramuscularly and half of the initial dose was used as the maintenance dose when deemed necessary. All surgical procedures were performed while the rabbits were breathing spontaneously. After the induction of anesthesia, the inguinal region was shaved for skin cleansing and then the skin and the field were cleaned using 10% povidone-iodine. Subsequent procedures were performed depending on the assigned group of animals, as follows - control group: no intervention was performed; sham group: after inguinal incision, only the ilioinguinal nerve was exposed by an appropriate dissection by an inguinal ligament-guided approach (Figure 1A); heavyweight mesh group: after inguinal incision, the ilioinguinal nerve was exposed and a heavyweight mesh (1 × 1 cm Prolene®, Ethicon, Somerville, NJ) was implanted on the nerve (Figure 1B); lightweight mesh group: after inguinal incision, the ilioinguinal nerve was exposed and a lightweight mesh (1 × 1 cm Ultrapro®, Ethicon, Somerville, NJ) was implanted on the nerve (Figure 1C). The meshes were fixed to the tissue on both sides with a 5/0 polypropylene suture. The skin was closed with 3/0 monofilament sutures in sham and both mesh groups.

After three months, all subjects were anesthetized with the same method described previously and the ilioinguinal nerve was exposed through the bilateral inguinal incision. In the control and sham groups, the ilioinguinal nerve was excised after ligating the distal and proximal ends. The ilioinguinal nerve was excised together with the mesh in the lightweight and heavyweight mesh groups. After collecting the samples, hemostasis was performed and the skin was closed with 3/0 monofilament sutures. The animals were not killed at the end of the experiment.

Histological tissue processing

The ilioinguinal nerve tissues were fixed in 2% phosphate-buffered glutaraldehyde and 10% paraformaldehyde solutions at pH 7.2 for four to six hours at 4 °C and underwent a post-fixation process in phosphate-buffered 1% osmium tetroxide. Tissue samples were dehydrated through a graded series of ethanol, transferred to propylene oxide, and embedded in Araldite® 6005 (Huntsman, Istanbul, Turkey). Semithin sections were cut in 800-1000 nm and stained with toluidin blue-azure II. All tissue fixation, embedding sectioning, and staining procedures were performed in the electron microscopy laboratory of the Department of Histology and Embryology, Ankara University Faculty of Medicine.

Morphometric analysis

For morphological analysis from semithin sections of samples, digital 8-bit RGB images were captured by a Leica ICC50 HD camera mounted on the light microscope (Leica DM 500) under a 100x magnification lens using immersion oil. A total of 50 randomly selected nerve fibers in each animal in control, sham, and mesh groups were analyzed using the Image J program [14]. The outer (fiber diameter) and inner diameter (axonal diameter) of selected nerve fibers were measured at two different locations (along the vertical and horizontal axes of the nerve fiber cross-section) using straight-line selection, and the mean values of the outer and inner diameter were obtained. Using the formula (axonal diameter/outer diameter) the G-ratio and the myeline thickness for each of the analyzed nerve fibers were calculated. The number of myelinated nerve fibers with a diameter ≤4 µm, 5-8 µm, and ≥9 µm were assessed by an experienced senior histologist blinded to groups.

Statistical analysis

Statistical analyses were performed using IBM SPSS Statistics v 15.0 (IBM Corp., Armonk, NY). The normality of the data was tested using the Kolmogorov-Smirnov and Shapiro-Wilk tests. Groups were compared using the Kruskal-Wallis test for non-normally distributed data. The post hoc multicomparison test was used to determine the groups yielding the statistical difference. The distribution of fiber diameters among the groups was analyzed using the Chi-squared test. The level of statistical significance was set at p<0.05.

## Results

**Figure 2:**
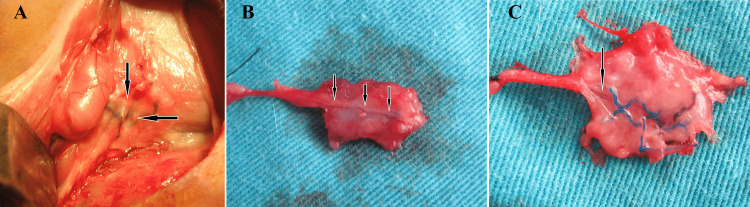
Exposure of the ilioinguinal nerve after three months (A) fibrous tissue around the mesh; (B) ilioinguinal nerve adhesion of the heavyweight mesh; (C) ilioinguinal nerve adhesion of the lightweight mesh. The arrows show the ilioinguinal nerve

None of the rabbits were deceased during or after the operation. During the three months of follow-up, no infective complications involving the surgical area were observed. In the sham group, no fibrous tissue was observed macroscopically around the ilioinguinal nerve re-exposed three months after the initial surgery. In the lightweight and heavyweight mesh groups, the inguinal region was very tight and there was similar dense fibrotic tissue around the mesh (Figure 2A), which showed adhesion to the nerve (Figures 2B, 2C).

Cytomorphological assessment

**Figure 3:**
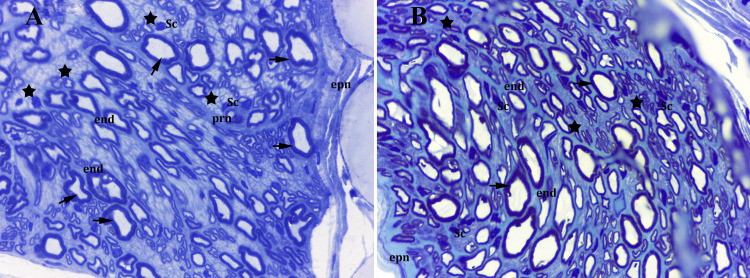
Semithin section of control and sham groups (A) control group; (B) sham group Arrows: myelinated fibers; stars: unmyelinated fibers; 100x magnification (oil immersion), toluidin blue-azure II Sc: Schwann cell nucleus; end: endoneurium; prn: perineurium; epn: epineurium

Histological examination of the control and sham groups by semithin sections of the peripheral nerve revealed that the myelinated nerve fibers were almost intact, showing normal morphology. The endoneurium between the nerve fibers also appeared to be normal (Figures 3A, 3B).

**Figure 4:**
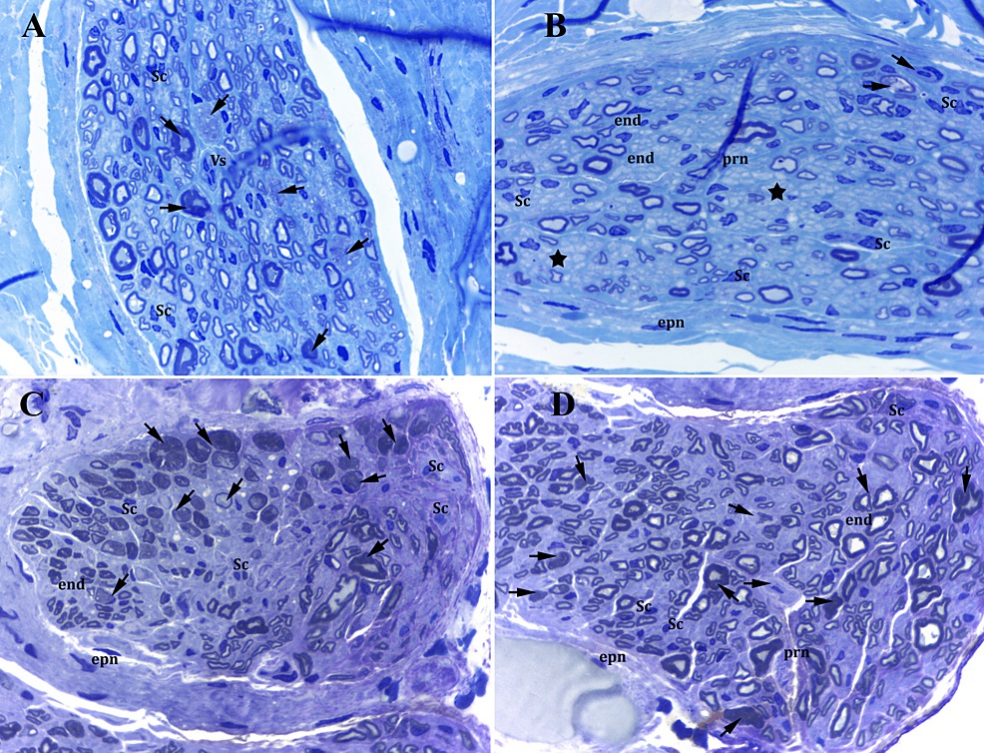
Semithin sections of mesh groups - 1 (A, B) lightweight mesh group. Arrows: vacuolization, the undulation of myelin layers, degenerated nerve fibers, and axons; stars: unmyelinated fibers; end: endoneurium with moderate edema; Vs: endoneurial blood vessels; Sc: Schwann cells (C, D) heavyweight mesh group. Arrows: degenerated myelinated nerve fibers were more prominent; stars: degenerated unmyelinated axons; Sc: Schwann cells; end: endoneurium; epn: epineurium 100x magnification (oil immersion), toluidin blue-azure II

**Figure 5:**
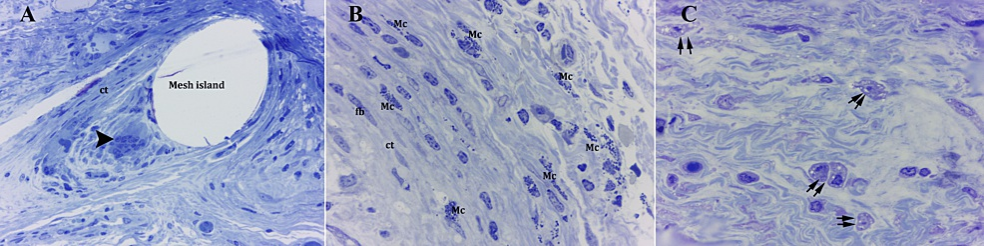
Semithin sections of mesh groups - 2 (A, B) heavyweight mesh group. Arrowhead: foreign body giant cells around mesh island; ct: connective tissue; Mc: mast cells with their granules; fb: fibroblasts (C) Lightweight mesh group. Double arrows: macrophages with their vacuolated cytoplasms 100x magnification (oil immersion), toluidin blue-azure II

Semithin sections of the ilioinguinal nerve in lightweight and heavyweight mesh groups exhibited grade I compression injury-induced morphologic changes [15]. In the lightweight mesh group, we observed a moderate increase in the number of the small-caliber, thinly myelinated fibers, which predominated in either the peripheral or central region of the nerve. Myelin sheaths of the nerve fibers showed undulation and vacuolization, and separation of the myelin layers as a prominent feature of myelin degeneration. Some fascicles showed a reduction in myelinated fiber density with axonal degeneration and mild endoneural edema (Figures 4A, 4B). In the heavyweight mesh group, nerve morphology exhibited more intense axonal and myelin degeneration and axonal dilatation, which was mostly exhibited in the peripheral regions of the nerve. Additionally, myelin sheath thickening, separation of the myelin layers, and myelin vacuolization were more pronounced compared to the lightweight mesh group. Also, we observed degenerative changes in the unmyelinated nerve fibers (Figures 4C, 4D). Connective tissue fibroblasts, macrophages with vacuolated cytoplasm, mast cells, and foreign body giant cells around mesh islands were observed in both lightweight and heavyweight mesh groups (Figures 5A, 5B, 5C).

The results of the morphometric assessment are presented in Table 1. There was no statistically significant difference between the control and sham groups in terms of any of the assessed parameters. The fiber diameter and the axon diameter were moderately increased in the heavyweight mesh group compared with the control, sham, and lightweight mesh groups (p<0.001, among all groups). The G-ratio was found in the normal range (0.50-0.70) within the control, sham, and both mesh groups. However, the G-ratio was moderately increased in the heavyweight mesh group compared to the control, sham, and lightweight mesh groups and the difference was statistically significant (p<0.001, among all groups). The myelin thickness was increased in the heavyweight mesh group and decreased in the lightweight mesh group (p<0.001).

**Table 1:** Morphometric assessment of the study groups Data are presented as mean ±standard deviation *P<0.001 in heavyweight mesh group vs. lightweight, sham, and control mesh groups. **P<0.001 in heavyweight mesh group vs. lightweight mesh group

Variables	Control (n=6)	Sham (n=6)	Lightweight mesh (n=10)	Heavyweight mesh (n=10)
Fiber diameter, µm	6.59 ±2.08	6.52 ±1.90	4.88 ±1.47	8.70 ±2.17*
Axon diameter, µm	3.77 ±1.62	3.73 ±1.41	2.89 ±1.17	5.76 ±1.79*
G-ratio	0.55 ±0.09	0.56 ±0.11	0.59 ±0.09	0.65 ±0.09*
Myelin thickness, µm	2.79 ±0.82	2.69 ±0.94	2.00 ±0.59	2.94 ±0.90**

The distribution of the fiber diameter in control, sham, and mesh groups is outlined in Table 2. The nerve fibers with a diameter of 5-8 µm were prominently observed in the control and sham groups (67% and 71%, respectively). The ratio of fibers with ≤4 µm diameter was found significantly higher in the lightweight mesh group than the other groups and the ratio of fibers with ≥9 µm diameter was found significantly higher in the heavyweight mesh group than the other groups (p<0.05). Small- and large-diameter nerve fiber distributions were similar in the control and sham groups (17% and 16% vs. 13% and 16%, respectively).

**Table 2:** Distribution of the fiber diameter ratio in the study groups *P<0.05 in lightweight mesh group vs. heavyweight, sham, and control groups. **P<0.05 in heavyweight mesh group vs. lightweight, sham, and control groups

Groups	≤4 µm	5-8 µm	≥9 µm
Control group	17%	67%	16%
Sham group	13%	71%	16%
Lightweight mesh group	47%*	51%	2%
Heavyweight mesh group	4%	42%	54%**

## Discussion

Foreign body reaction develops against meshes used for hernia repair and leads to the accumulation of mast cells, macrophages, giant-cell granulocytes, fibroblasts, and connective tissue collagen fibers. It has been demonstrated that a similar type of foreign body reaction occurs on the surfaces of different meshes [16]. Nevertheless, it is thought that foreign body response and intensity of inflammation are determined by the characteristics of biomaterials [17]. Various kinds of grafts are developed to enhance the integration of meshes and to reduce mesh-related foreign body reactions. The weight and pore size of biomaterials are among the characteristics discussed most frequently [17]. The meshes with lower weight and enlarged pores are associated with less foreign body reaction and inflammation, better accommodation with the surrounding tissues, and they incorporate better [18]. In a rat model, a time-dependent decrease was observed in the mast cell infiltration at the tissue-mesh interface with the lightweight mesh, and mast cell response was predominant in the heavyweight mesh [19]. Another study has suggested that the absorbable poliglecaprone component of the lightweight mesh might lead to an inflammatory reaction in the early period and that the disappearance of this inflammatory reaction with the dissolution of the absorbable component could result in less passive compression of the lightweight mesh [20]. In our study, the development of fibrotic tissue around the mesh and adhesion of the mesh to the ilioinguinal nerve were observed on macroscopic evaluation. Giant cells, macrophages, and mast cells were observed in the semithin sections of the mesh groups, suggesting a foreign body reaction.

Several studies have examined the relationship between mesh characteristics and the incidence of postoperative chronic pain and recurrence. In a randomized clinical trial comparing lightweight and heavyweight meshes, O'Dwyer et al. reported that chronic pain decreased and recurrence increased in the lightweight mesh at one-year follow-up after open inguinal hernia repair [21] However, other studies have reported no difference in postoperative pain or recurrence at long-term follow-ups after the use of either type of mesh [22]. Bringman et al. have reported that the use of lightweight mesh in Lichtenstein hernia repair did not affect recurrence rates, but improved pain and groin discomfort [23]. Some studies have shown that the lightweight mesh is not superior to the heavyweight mesh in terms of chronic pain, but it has been emphasized that groin discomfort and foreign body feeling are less commonly associated with it [24,25]. Postoperative chronic inguinal pain may be due to entrapment or tension on the nerves. Inguinal nerve (ilioinguinal, iliohypogastric, and genitofemoral) injury during open hernia repair can occur due to several reasons such as partial or complete nerve transection, neuroma formation, cautery-related injury, stretching, crushing, suture compression, and mesh-related entrapment. Our results suggest that the ilioinguinal nerve was affected by the fibrosis process caused by two types of meshes. Studies investigating the direct effect of the mesh and associated fibrotic reaction on the inguinal nerves are scarce. In a study evaluating the mesh-related ilioinguinal nerve entrapment, granulomatous infiltrate and loss of myelinated axons were demonstrated and the cause of chronic neuropathic pain was attributed to the neuropathic alterations secondary to mesh-related inflammation [26]. In our study, we have demonstrated nerve injury in both mesh groups secondary to compression caused by the mesh-related fibrotic reaction, and unmyelinated axonal injury was more pronounced in the heavyweight mesh group than the lightweight mesh group. Demirer et al. have proposed that chronic pain after hernia repair may be caused by the entrapment of the nerve in the scar tissue induced by the polypropylene mesh [27].

The G-ratio, which is used as an index of optimal myelinization for the conduction velocity of nerve impulses, reflects axonal function and structural integrity [28]. In peripheral nerves, the G-ratio (axon diameter to total fiber diameter) is between 0.50 and 0.70 for optimal nerve conduction in most of the myelinated fibers [29]. In our study, we found that the G-ratio was moderately increased in the heavyweight mesh group compared to the lightweight mesh and other groups. Axon and fiber diameter were reduced in the lightweight mesh group and increased in the heavyweight mesh group compared to the control and sham groups. The ratio of myelinated axons with a diameter ≥9 µm was highest (54%) in the heavyweight mesh group, which may indicate axonal dilatation and degeneration. This finding may be attributed to the intensity of the compression related to the weight of the mesh and concurrent fibrosis. Small-diameter nerve fibers (≤4 µm) were predominantly observed in the lightweight mesh group, which may represent nerve regeneration and indicate lower levels of injury by the lightweight mesh as they appear earlier in the lightweight group than in the heavyweight group.

This study has a few limitations. Our major limitation was the small sample size of the study. To overcome this limitation, we used both inguinal regions of the subjects to increase the number of tissues analyzed. The second limitation was that the study did not report on the extent of fibrosis, including extracellular matrix components and fibroblasts surrounding the ilioinguinal nerve. Another limitation of our study is the fact that mesh implantation is not performed directly on the ilioinguinal nerve during anterior open hernia repair in daily practice. However, due to anatomical variations of the nerve or injury of the protective fascia of the nerve, the mesh may have direct contact with the nerve. Another limitation was the inability to evaluate chronic pain. Pain scales developed for animals are used in the assessment of acute pain [30]. Since this was an experimental animal study, we could not draw definite conclusions that these histopathological and morphological findings in the ilioinguinal nerves would necessarily cause postoperative chronic pain.

## Conclusions

This study suggests that both types of meshes induce cytomorphological alterations on the adjacent nerve tissues caused by foreign body reaction and compression. Our results also suggest that myelin sheath thickening, separation of the myelin layers, and myelin vacuolization were more pronounced in the heavyweight mesh compared to the lightweight mesh. Additionally, the G-ratio was moderately increased in the heavyweight mesh group compared to the lightweight mesh group. We found that ilioinguinal nerve degeneration was higher in the heavyweight mesh group than in the lightweight mesh group. We think that histological alterations on the ilioinguinal nerves caused by different meshes may be related to chronic pain and groin discomfort after hernia surgery and our study may serve as a guide for future studies in the field.

## References

[1] Jenkins JT, O'Dwyer PJ (2008). Inguinal hernias. BMJ.

[2] Alvarez JA, Baldonedo RF, Bear IG, Solís JA, Alvarez P, Jorge JI (2004). Incarcerated groin hernias in adults: presentation and outcome. Hernia.

[3] Öberg S, Andresen K, Rosenberg J (2017). Absorbable meshes in inguinal hernia surgery: a systematic review and meta-analysis. Surg Innov.

[4] Nilsson H, Angerås U, Sandblom G, Nordin P (2016). Serious adverse events within 30 days of groin hernia surgery. Hernia.

[5] Lundström KJ, Sandblom G, Smedberg S, Nordin P (2012). Risk factors for complications in groin hernia surgery: a national register study. Ann Surg.

[6] Bringman S, Conze J, Cuccurullo D (2010). Hernia repair: the search for ideal meshes. Hernia.

[7] Klosterhalfen B, Junge K, Klinge U (2005). The lightweight and large porous mesh concept for hernia repair. Expert Rev Med Devices.

[8] Poobalan AS, Bruce J, Smith WC, King PM, Krukowski ZH, Chambers WA (2003). A review of chronic pain after inguinal herniorrhaphy. Clin J Pain.

[9] Fränneby U, Sandblom G, Nordin P, Nyrén O, Gunnarsson U (2006). Risk factors for long-term pain after hernia surgery. Ann Surg.

[10] Haladu N, Alabi A, Brazzelli M, Imamura M, Ahmed I, Ramsay G, Scott NW (2022). Open versus laparoscopic repair of inguinal hernia: an overview of systematic reviews of randomised controlled trials. Surg Endosc.

[11] Mongelli F, Ferrario di Tor Vajana A, FitzGerald M (2019). Open and laparoscopic inguinal hernia surgery: a cost analysis. J Laparoendosc Adv Surg Tech A.

[12] Aasvang EK, Brandsborg B, Christensen B, Jensen TS, Kehlet H (2008). Neurophysiological characterization of postherniotomy pain. Pain.

[13] Council of Europe (1986) (2023). Council of Europe (1986): European Convention for the Protection of Vertebrate Animals Used for Experimental and Other Scientific Purposes - European Treaty Series No 123. European Treaty Series No 123 Accessed 20 April 2020.

[14] Collins TJ (2007). ImageJ for microscopy. Biotechniques.

[15] Sunderland S (1951). A classification of peripheral nerve injuries producing loss of function. Brain.

[16] Klosterhalfen B, Klinge U, Hermanns B, Schumpelick V (2000). Pathology of traditional surgical nets for hernia repair after long-term implantation in humans (Article in German). Chirurg.

[17] Weyhe D, Belyaev O, Müller C, Meurer K, Bauer KH, Papapostolou G, Uhl W (2007). Improving outcomes in hernia repair by the use of light meshes--a comparison of different implant constructions based on a critical appraisal of the literature. World J Surg.

[18] Cobb WS, Kercher KW, Heniford BT (2005). The argument for lightweight polypropylene mesh in hernia repair. Surg Innov.

[19] Rosch R, Junge K, Schachtrupp A, Klinge U, Klosterhalfen B, Schumpelick V (2003). Mesh implants in hernia repair. Inflammatory cell response in a rat model. Eur Surg Res.

[20] Cobb WS, Burns JM, Peindl RD, Carbonell AM, Matthews BD, Kercher KW, Heniford BT (2006). Textile analysis of heavy weight, mid-weight, and light weight polypropylene mesh in a porcine ventral hernia model. J Surg Res.

[21] O'Dwyer PJ, Kingsnorth AN, Molloy RG, Small PK, Lammers B, Horeyseck G (2005). Randomized clinical trial assessing impact of a lightweight or heavyweight mesh on chronic pain after inguinal hernia repair. Br J Surg.

[22] Demetrashvili Z, Khutsishvili K, Pipia I, Kenchadze G, Ekaladze E (2014). Standard polypropylene mesh vs lightweight mesh for Lichtenstein repair of primary inguinal hernia: a randomized controlled trial. Int J Surg.

[23] Bringman S, Wollert S, Osterberg J, Smedberg S, Granlund H, Heikkinen TJ (2006). Three-year results of a randomized clinical trial of lightweight or standard polypropylene mesh in Lichtenstein repair of primary inguinal hernia. Br J Surg.

[24] Yazdankhah Kenary A, Afshin SN, Ahmadi Amoli H (2013). Randomized clinical trial comparing lightweight mesh with heavyweight mesh for primary inguinal hernia repair. Hernia.

[25] Rutegård M, Gümüsçü R, Stylianidis G, Nordin P, Nilsson E, Haapamäki MM (2018). Chronic pain, discomfort, quality of life and impact on sex life after open inguinal hernia mesh repair: an expertise-based randomized clinical trial comparing lightweight and heavyweight mesh. Hernia.

[26] Miller JP, Acar F, Kaimaktchiev VB, Gultekin SH, Burchiel KJ (2008). Pathology of ilioinguinal neuropathy produced by mesh entrapment: case report and literature review. Hernia.

[27] Demirer S, Kepenekci I, Evirgen O (2006). The effect of polypropylene mesh on ilioinguinal nerve in open mesh repair of groin hernia. J Surg Res.

[28] Stahon KE, Bastian C, Griffith S, Kidd GJ, Brunet S, Baltan S (2016). Age-related changes in axonal and mitochondrial ultrastructure and function in white matter. J Neurosci.

[29] Jortner BS (2011). Preparation and analysis of the peripheral nervous system. Toxicol Pathol.

[30] Mich MP, Hellyer WP (2009). Objective, categoric methods for assessing pain and analgesia. Handbook of Veterinary Pain Management.

